# Alcohol and fertility: how much is too much?

**DOI:** 10.1186/s40738-017-0037-x

**Published:** 2017-07-10

**Authors:** Kristin Van Heertum, Brooke Rossi

**Affiliations:** Department of Reproductive Biology - Case Western Reserve University School of Medicine, University Hospitals MacDonald Women’s Hospital, 11100 Euclid Avenue, Cleveland, OH 44106 USA

**Keywords:** Alcohol, Infertility, Fertility, Lifestyle, Fecundability

## Abstract

Alcohol use is prevalent in the United States. Given that a substantial portion of the drinking population is of reproductive age, it is not uncommon for couples who are attempting conception, or for women who are already pregnant, to be regularly consuming alcohol. Alcohol use is associated with multiple reproductive risks, including having a child with a Fetal Alcohol Spectrum Disorder, increased risk of fetal loss, and decreased chance of live birth. This review serves to examine the risks of alcohol in the context of reproductive health.

## Background

Approximately 12% of couples in the U.S. experience difficulty conceiving or impaired fecundity, defined as the ability to achieve a live birth in a single menstrual cycle [1]. As alcohol is the most widely used recreational substance, it is important to understand any deleterious effects it has on human reproduction [2]. In this review, we will discuss the prevalence of alcohol use in the U.S.; the health risks and benefits associated with alcohol consumption outside of reproduction; the risks of alcohol use in pregnancy including congenital anomalies and pregnancy loss; the effects of alcohol on fertility in both women and men, such as alcohol’s impact on ovarian reserve, steroid hormone production, sperm quality and fecundability; and finally the impact of alcohol consumption on fertility treatments.

### Prevalence of alcohol use and abuse

Alcohol use is common in the United States. The 2015 National Survey on Drug Use and Health (NSDUH) found that 86.4% of people age 18 or older reported alcohol consumption at some point in their lives, and 56% reported drinking in the past month [3]. The survey reported a prevalence of binge drinking, defined as drinking a quantity of alcohol to raise the blood alcohol concentration (BAC) to 0.08 g/dL (typically 4 drinks for women and 5 drinks for men in 2 h), of 26.9% (see Table 1 for alcohol consumption definitions) [4]. Another study performed using data from the Behavioral Risk Factor Surveillance System (BRFSS), a telephone based survey implemented by U.S. state health departments, found that while the overall prevalence of alcohol consumption is not increasing, it appears that the rate of binge drinking is rising across the country [5].

**Table 1:** Definitions of Levels of Alcohol Consumption (compiled from [7])

Level of consumption	Definition
Current use	≥ 1 drink in the past 30 days
Moderate use	Up to 1 drink per day for women, up to 2 drinks per day for men
Binge drinking	Drinking a quantity of alcohol to raise the BAC to 0.08 g/dL - typically 4 drinks for women and 5 drinks for men in 2 h
Heavy alcohol use	Binge drinking on ≥5 days in the past month

The rates of alcohol use in pregnancy in the U.S. remain surprisingly high. According to a report from the Substance Abuse and Mental Health Services Administration, 8.5% of pregnant women in 2011-2012 reported current alcohol use, 2.7% reported binge drinking, and 0.3% reported heavy drinking, defined as 5 more episodes of binge drinking in the past month [6, 7]. A recent cohort study of over 5000 pregnant women found that women with an intended pregnancy were 31% less likely to consume alcohol in pregnancy than those with unintended pregnancies [8]. This study also found several surprising characteristics associated with drinking in pregnancy, including college-education, white race, older age (particularly over 35 years), higher income, and nulliparity. Factors associated with binge drinking during pregnancy in this study were smoking (past or current), illicit drug use, younger age and being unmarried. Other risk factors for continuation of alcohol use during pregnancy include stressful life events prior to conception and a high level of pre-pregnancy alcohol consumption [9, 10]. Women may be less likely to drink during pregnancy if they have experienced any difficulty conceiving [10].

Rates of alcohol use in women undergoing fertility treatment vary across different studies, but appear to be somewhere between 26 and 41% [11, 12]. However, studying alcohol consumption in a group of women who are attempting conception or are already pregnant presents significant challenges. While recall bias can occur in any population, these women may be less likely to accurately report their level of alcohol consumption as they may be embarrassed by, or feel guilty about, their alcohol use.

### Non-reproductive sequelae of alcohol use

Excessive alcohol intake can lead to multiple chronic diseases including hypertension, heart disease, liver disease, gastrointestinal bleeds, cancer (breast, mouth, throat, esophagus, liver, colon), dementia and other cognitive deficits, anxiety/depression, and social and economic losses, such as damage to relationships and loss of employment [13]. Conversely, moderate alcohol intake, defined as up to 1 drink per day for women and up to 2 drinks per day for men, may offer some health benefits [14, 15]. These benefits include decreased risk of stroke and diabetes, as well as decreased risk of heart disease or mortality from heart disease. In 2005, it was estimated that 26,000 deaths were prevented in the U.S. due to reductions in ischemic heart disease, diabetes and ischemic stroke because of benefits attributed to moderate alcohol consumption [6]. However, care providers must still balance the overall risks and benefits of alcohol use when counseling their patients on their level of alcohol intake.

### Alcohol use during pregnancy

The teratogenic effects of alcohol use during pregnancy are well documented [16]. Alcohol readily crosses the placenta to the amniotic fluid and fetus [17]. The fetus will typically be exposed to higher concentrations of alcohol than the mother due to accumulation of alcohol and its metabolites in the amniotic fluid, and comparatively reduced fetal metabolic enzyme activity [17]. Some proposed mechanisms of teratogenicity include impaired anti-oxidant capability, increased free radicals and reactive oxygen species with resultant increased apoptosis in fetal cranial/brain tissue [17].

Fetal Alcohol Spectrum Disorders (FASD), which are caused by alcohol exposure in utero, include fetal alcohol syndrome (FAS), partial fetal alcohol syndrome (PFAS), alcohol-related neurodevelopmental disorder (ARND) and alcohol-related birth defects (see Table 2 for summary of characteristics) [18]. FASD represents a continuum of disease characterized by behavioral and cognitive deficits, craniofacial anomalies, and growth retardation. Prevalence of FASD has been estimated at 2-5% in the general U.S. population, with rates of FAS estimated to be 0.2 to 7 per 1,000 children [19]. While studies have shown that the degree of deficits/defects worsens with increasing dose and exposure time, there has been no definitive identification of a safe exposure dose or duration in pregnancy [20, 21]. A recent prospective cohort of 992 women found a strong association between consumption of alcohol in the late first trimester and some characteristic facial anomalies, microcephaly, low birth weight and reduced length [22]. However, alcohol use in the second trimester was also associated with low birth weight and length, while use in the third trimester only effected birth length. Other studies have confirmed that growth deficiency, neurobehavioral issues and microcephaly can occur following alcohol exposure in any trimester, but the characteristic facial features are likely due to first trimester exposure [23]. In many studies, it is often difficult to determine if alcohol was consumed in an isolated trimester or throughout the pregnancy. Therefore, it is not possible, currently, to make a determination regarding the fetal effects of alcohol in women who abstain from use in the first and/or second trimesters and subsequently use alcohol in the third trimester.

**Table 2:** Fetal Alcohol Spectrum Disorders – all diagnoses require documented prenatal alcohol exposure (compiled from [19])

Disorder	Features
Fetal alcohol syndrome	Facial dysmorphia, growth deficits, and CNS abnormality
Partial fetal alcohol syndrome	Facial dysmorphia with growth deficits or CNS abnormality
Alcohol related neurodevelopmental disorder	CNS abnormality and/or intellectual disabilities without growth deficits or facial dysmorphia
Alcohol related birth defects	Facial dysmorphia plus additional birth defect(s) without growth deficits or CNS abnormality

There is conflicting data regarding the effects of alcohol exposure in utero when there is no evidence for FASD. Several studies from the Danish National Birth Cohort did not identify any effect on general intelligence, attention or executive function in 5 year old children whose mothers reported low-consumption, moderate-consumption, or binge drinking compared with children whose mothers reported no alcohol use in pregnancy [24, 25]. However, there are weaknesses to these studies, as they did not include any diagnostic evaluation for FASD in their cohort, and 5 years of age may be too young to make a true assessment on any neuropsychological effects of alcohol, as the brain is still developing at this age [26].

The findings of studies examining the effects of alcohol intake on the risks of pregnancy loss have been variable [27]. This, in part, can be attributed to the inconsistency of classification of alcohol consumption: some studies report on a dichotomous categorization of use or no use, while others include information on specifics of amount or type of alcohol used. Additionally, given the clear documentation of the teratogenicity of alcohol, this is not a subject that allows for a robust study such as a randomized controlled trial. Finally, as mentioned previously, if women think it is socially unacceptable to drink alcohol while pregnant, they may underreport or not report use.

There is some consensus that at a threshold of 2 to 4 drinks per week the risk of miscarriage begins to increase, particularly in early pregnancy, though there have been several studies that did not document any increased risk of fetal loss with any level of alcohol consumption [28–30]. Table 3 provides a summary of notable findings on fetal loss. It has been theorized that an increase in reactive oxygen species plays a significant role in the pathogenesis of fetal loss due to alcohol exposure [31]. Avalos et al., in a prospective cohort study in the Kaiser Permanente system, found that women who consumed 4 or more alcoholic beverages per week were more than twice as likely to experience a miscarriage as those who did not drink any alcohol (HR 2.65, 95% CI 1.38-5.10) [27]. The study did not find any increased risk of miscarriage in those women who drank less than 4 drinks per week, or in women who drank only beer or wine, compared to those who abstained. The study did, however, document a significantly increased risk of fetal loss in women who only drank liquor compared to those who did not drink at all (HR 2.24, 95% CI 1.32-3.80). Another study from the Danish health registry had similar findings, with the risk of first trimester loss in those women who drank 4 or more drinks per week being more than double the risk of those who abstained (HR 2.82, 95% CI 2.27-3.49) [28]. This study also found that women who consumed 2-3.5 drinks per week had an increased risk of miscarriage in the first trimester (HR 1.66, 95% CI 2.27–3.49) as well as fetal loss at 13–16 weeks (1.57, 95% CI 1.30-1.90). A different Danish cohort study also documented an increase in the risk of stillbirth in those who consumed 5 or more drinks per week in pregnancy, versus those who drank less than one drink per week on average (OR 2.65, 95% CI 1.18-5.97) [32]. The study did not find any increase in the risk of neonatal death with any amount of alcohol consumption in pregnancy. On the other hand, pre-pregnancy alcohol consumption, at least in low to moderate amounts, does not appear to increase the risk of miscarriage or stillbirth [33]. The recommendation, therefore, should be for pregnant women to abstain from any alcohol use in pregnancy, as even those women who drink less than moderately are at an increased risk for loss, in addition to the risk of FASD with even low doses of alcohol exposure.

**Table 3:** Summary of study findings on alcohol and pregnancy loss

Level of alcohol consumption in pregnancy	Effects on pregnancy loss	Reference
Any alcohol consumption vs. abstaining	No increased risk of miscarriage (RR 1.1, 95% CI 0.9 - 1.4)	Parazzini, et al. 1994
≥ 4 drinks per week vs. abstaining	Increased risk of miscarriage (HR 2.65, 95% CI 1.38 - 5.10)	Avalos, et al. 2014
	Increased risk of miscarriage (HR 1.66, 95% CI 2.27 - 3.49) Increased risk of 13-16 week loss (HR 1.57, 95% CI 1.30 - 1.90)	Andersen, et al. 2012
≥ 5 drinks per week vs. < 1 drink per week	Increased risk of stillbirth (OR 2.65, 95% CI 1.18 - 5.97)	Kesmodel, et al. 2002

### Effects of alcohol on female reproduction

The physiologic effects of alcohol consumption on female reproductive physiology have not been well delineated due to a paucity of high quality studies in this area. Table 4 summarizes several of the studies reviewed below. Studies in humans and animal models have found alterations in ovulation and menstrual cycle regularity with chronic/prolonged alcohol intake, though amount consumed is often not specified [34]. Schliep et al. found that acute alcohol use increased estradiol, testosterone and LH levels, with greater increases seen in women who reported recent binge drinking, though with no associated menstrual cycle dysfunction [35]. While acute alcohol consumption may have little or no associated effect on the menstrual cycle, there does appear to be a negative effect on fertility treatment outcomes, as will be discussed later.

**Table 4:** Summary of study findings on alcohol and female reproductive function

Level of alcohol consumption	Effects on female reproduction	Reference
> 1 drink per day vs. abstaining	No increased risk of ovulatory infertility (after controlling for confounders)	Chavarro, et al. 2009
1-3 drinks per week vs. abstaining	No difference in adjusted fecundability	Mikkelsen, et al. 2016
4-7 drinks per week vs. abstaining		
8-13 drinks per week vs. abstaining		
≥ 14 drinks per week vs. abstaining		
1-5 drinks per week vs. abstaining	Decreased chance of clinical pregnancy (OR 0.61, 95% CI 0.4 - 0.93)	Jensen, et al. 1998
> 10 drinks per week vs. abstaining	Decreased chance of clinical pregnancy (OR 0.34, 95% CI 0.22 - 0.52)	
Low consumers (< 50 g per week) vs. Moderate consumers (50 - 140 g per week) vs. High consumers (> 140 g per week)^a^	Increased risk of seeking fertility treatment with increasing alcohol intake: High vs. moderate RR 1.58 (95% CI 1.07 - 2.34) Low vs. high RR 0.64 (95% CI 0.46 - 0.90)	Eggert, et al. 2004
1-6 drinks per week vs. < 1 drink per week (in women over age 30)	Increased incidence of infertility (Adjusted HR 1.95, 95% CI 1.04 - 3.66)	Tolstrup, et al. 2003
Binge drinking ≥2 times per week vs. drinkers who do not binge	26% lower AMH level (*p* < 0.04)	Hawkins, et al. 2016

^a^One standard drink in the U.S. has roughly 14 g of alcohol [65]

Heavy alcohol use may diminish ovarian reserve and fecundability in women. Ovarian reserve, a measure of a woman’s reproductive potential determined by her remaining oocytes, can be measured in a variety of ways, including serum follicle stimulating hormone (FSH) and anti-Müllerian hormone (AMH) levels as well as antral follicle count [36]. A study of African American women in Michigan found that women who regularly binge drink two or more times a week had a 26% lower AMH level than current drinkers who do not binge after age-adjustment [37]. There is also evidence that women who suffer from alcoholism may experience menopause at an earlier age than their non-alcoholic counterparts [38].

On the other hand, the relationship between light to moderate alcohol use and female infertility has yet to be fully characterized [39]. An 8-year cohort study of 18,555 women without a history of infertility who were attempting to conceive found no relationship between alcohol consumption and ovulatory dysfunction [40]. Multiple other studies have found no relationship between moderate alcohol consumption and fecundability [41–43]. A retrospective study of almost 40,000 pregnant women actually reported a shortened time to pregnancy in women who consumed a moderate amount of alcohol compared with those who did not drink at all [44]. However, a Danish cohort study found that, compared with women who drank no alcohol, women who reported consuming 1–5 drinks per week, in addition to those who consumed more than 10 drinks per week, had a decreased chance of achieving a clinical pregnancy (OR 0.61, 95% CI 0.40-0.93 and OR 0.34, 95% CI 0.22-0.52, respectively) [45]. A cohort survey-based study of 7,393 Swedish women also found a dose-response relationship of the amount of alcohol consumed to the risk of seeking treatment for infertility, with high alcohol consumers being more likely to seek treatment than moderate drinkers (RR 1.58, 95% CI 1.07–2.34), while low consumers had a significantly lower risk of pursuing fertility treatment (RR 0.64, 95% CI 0.46-0.90) [46]. Another study from Denmark found that alcohol intake of 1–6 drinks per week in women over the age of 30 may be associated with an increased incidence of infertility when compared to women of the same age who consume less than one drink per week [47]. Though the findings are inconsistent, women who are already seeking treatment for infertility should be encouraged to minimize alcohol consumption, as even moderate levels could negatively impact their ability to conceive.

### Effects of alcohol on male reproduction

Alcohol consumption in men can also cause difficulties with fertility. Some studies on long-term, heavy alcohol use have reported reduced gonadotropin release, testicular atrophy, and decreased testosterone and sperm production [48]. Other studies of men who drink heavily have documented increases in gonadotropins and estradiol, independent of liver disease, with decreased testosterone as a consistent finding [49]. Alcoholism is also associated with liver dysfunction, which can result in hormonal disturbances due to the inability to metabolize estrogens. A decrease in the quality of semen parameters has also been consistently documented in heavy consumers of alcohol, even with occasional azoospermia [50]. Furthermore, it has been well documented that alcohol abuse and acute intoxication are associated with sexual dysfunction, including issues with arousal and desire, as well as erectile and ejaculatory dysfunction, all of which could lead to difficulties conceiving if men are unable to have effective intercourse [48, 49, 51].

The effects of low to moderate consumption of alcohol, however, do not appear to be clinically significant [21, 52]. Table 5 provides a summary of several of the studies cited here. Multiple studies have found a decrease in normal sperm morphology in men who regularly drink alcohol, with no other associated alterations in semen parameters [49]. Two large cohort studies failed to identify a correlation between male alcohol consumption and fecundability [53, 54]. A cross-sectional study of over 8,000 men from the U.S. and Europe who were classified as low to moderate consumers of alcohol found no difference in semen parameters, and actually documented a linear increase in serum testosterone levels with increasing amounts of alcohol consumption [55]. Several other studies have similarly shown no effect in semen parameters with moderate alcohol consumption [56, 57]. Therefore, men who drink heavily should be advised to decrease their alcohol intake. However, those who drink moderately should be counseled regarding alcohol consumption based on their overall health status, and not necessarily on reproductive health.

**Table 5:** Summary of study findings on alcohol and male reproductive function

Level of alcohol consumption	Effects on male reproduction	Reference
Any alcohol consumption	No effect on fecundability	Curtis, et al. 1997
	No increased subfecundity	Olsen, et al. 1997
	No effect on any semen parameters or pregnancy rate	de Jong, et al. 2014
	No difference in any semen parameters	Jensen, et al. 2014
> 20 drinks per week vs. 1-10 drinks per week	Increased serum free testosterone (19.7-24.6 pmol/l higher) and total testosterone (0.9-1.0 nmol/l higher)	

### Effects on infertility treatment

There is substantial evidence that alcohol use, even in moderate quantities, negatively affects assisted reproductive technology (ART) outcomes [58]. A multicenter prospective study of 221 couples undergoing IVF or gamete intrafallopian transfer (GIFT) found a 13% decrease in the number of oocytes retrieved (95% CI -2% to −23%), a 2.86 times higher chance of not achieving pregnancy (95% CI 0.99–8.24) and a 2.21 times higher risk of miscarriage (95% CI 1.09 to 4.49) when the woman consumed one additional drink per day compared to those who had one less in the weeks before treatment [11]. The study also found a higher risk of not achieving a live birth when men drank alcohol in the month leading up to the treatment cycle, particularly when men drank the week of the sperm collection (OR 8.32, 95% CI 1.82–37.97). Another study of 2,545 couples undergoing 4,729 cycles of in vitro fertilization (IVF) examined the effects of variable amounts of alcohol consumption at the time of initiation of IVF stimulation [12]. The study found a decreased rate of live birth in women who consumed 4 or more drinks per week compared with those who drank less than 4 drinks per week (OR 0.84, 95% CI 0.71-0.99). In couples in which both the man and the woman drank 4 or more alcohol beverages per week, the live birth rate was decreased even further compared to those couples in which both partners drank less than 4 drinks per week (OR 0.79, 95% CI 0.66-0.96). These findings were largely felt to reflect failures in fertilization. Therefore, as it appears that even moderate levels of alcohol intake can decrease success with IVF by decreasing oocyte yield and live birth rates, efforts should be made to decrease alcohol use prior to initiating treatment with IVF.

The etiology for the detrimental effects on IVF outcomes has not been identified. However, as previously mentioned, acute alcohol consumption can cause increases in estradiol, testosterone, and LH levels [35]. Furthermore, estrogens are metabolized by the liver, and FSH is cleared by the kidneys and the liver [59, 60]. Therefore, alterations in liver function due to alcohol consumption may result in altered metabolism of the exogenous gonadotropins used in IVF, as well as the estrogen response of ovarian follicles to stimulation. In theory, these hormonal shifts could result in abnormal folliculogenesis and impaired endometrial receptivity.

The effects of alcohol on other forms of fertility treatments have not been well studied. One trial of 932 couples randomized to natural cycle with intracervical insemination (ICI), controlled ovarian stimulation (COS) with ICI, natural cycle with intrauterine insemination (IUI) or COS with IUI examined the effects of multiple lifestyle factors [61]. The study found that across all treatment groups, the pregnancy and live birth rates were higher in women who reported past alcohol usage (previously consumed at least one alcoholic beverage per week more than a month ago) than in current users or those that reported never consuming alcohol. However, this study did not further stratify alcohol usage by amount, and therefore it is difficult to extrapolate this data to form any recommendations.

## Conclusion

Given the potentially devastating consequence of FASD, women who are pregnant, attempting to conceive, or at risk for unintended pregnancy should be screened for alcohol use. The women should also be advised against consuming any amount of alcohol, as no “safe dose” has been identified, and effects to the fetus may begin as early as immediately after implantation [2, 62]. Furthermore, ART should not be provided for women who are unwilling or unable to minimize their consumption of alcohol [63]. Women are more likely to abuse alcohol if they are unsuccessful in conceiving after initial infertility evaluation, so continued screening for alcohol use should be performed throughout treatment [64]. Those women who do undergo ART should be advised to minimize their alcohol consumption prior to initiating treatment, as even moderate amounts of alcohol may decrease their chances of a successful live birth. While a moderate level of drinking does not appear to alter outcomes in men, male partners should be advised to at least avoid alcohol the week before they provide a semen sample for IVF.

## Abbreviations

AMH: Anti-Müllerian hormone
ARND: Alcohol-related neurodevelopmental disorder
ART: Assisted reproductive technology
BAC: Blood alcohol concentration
BRFSS: Behavioral Risk Factor Surveillance System
COS: Controlled ovarian stimulation
FAS: Fetal alcohol syndrome
FASD: Fetal alcohol spectrum disorders
FSH: Follicle stimulating hormone
GIFT: Gamete intrafallopian transfer
ICI: Intracervical insemination
IUI: Intrauterine insemination
IVF: In vitro fertilization
NSDUH: National Survey on Drug Use and Health
PFAS: Partial fetal alcohol syndrome
